# Single‐Cell Transcriptomic Analysis of Chemotherapy‐Induced Changes in Osteosarcoma With a Pyroptosis‐Related Gene‐Based Prognostic Model

**DOI:** 10.1111/jcmm.71110

**Published:** 2026-03-27

**Authors:** Tao Jin, Lei Dong, Wang Kai, Ziyang Yu, Guoyong Yu, Weifeng Liu

**Affiliations:** ^1^ Department of Orthopaedic Oncology Surgery Beijing Jishuitan Hospital, Capital Medical University, National Center for Orthopaedics Beijing China; ^2^ Beijing Research Institute of Traumatology and Orthopaedics Beijing China; ^3^ Department of Breast Disease Xiyuan Hospital of China Academy of Chinese Medical Sciences Beijing China; ^4^ Department of Pain Medicine Center Peking University Third Hospital Beijing China; ^5^ Beijing University of Chinese Medicine Beijing Beijing China; ^6^ Department of Osteoarthritis Trauma Hanzhong Central Hospital Hanzhong China

## Abstract

Osteosarcoma, the most common primary malignant bone tumour, presents significant treatment challenges due to its complex tumour microenvironment and the development of chemoresistance. This study employs single‐cell transcriptomics to investigate chemotherapy‐induced changes in osteosarcoma at both the cellular and molecular levels. Single‐cell RNA sequencing data were analysed to identify cell subpopulations and their responses to chemotherapy. Differential gene expression and pathway enrichment analyses were performed to elucidate chemotherapy‐induced changes. Additionally, we developed and validated a predictive model based on pyroptosis‐related genes, named Pyroscore, using 101 different machine‐learning algorithms. Chemotherapy led to an increased proportion of osteoclasts, endothelial cells, mesenchymal stem cells and pericytes, while decreasing T and NK cells, B cells, chondroblasts, monocytes and macrophages. Chemotherapy markedly upregulates the pyroptosis pathway in tumour cells, suggesting that chemotherapy induces programmed cell death in cancer cells through the activation of pyroptosis. Metabolic pathway analysis revealed significant inhibition of sulphur metabolism, starch and sucrose metabolism, pentose phosphate pathway, inositol phosphate metabolism, nitrogen metabolism and fatty acid metabolism. The Pyroscore model, which incorporates BAK1, CASP1, CASP5 and CASP6, demonstrated robust prognostic value across multiple data sets, with high scores correlating with improved survival outcomes. This study highlights the impact of chemotherapy on osteosarcoma cell subpopulations and the tumour microenvironment. The activation of the pyroptosis pathway and the development of the pyroscore prognostic model provide new insights into the mechanisms of chemotherapy response and potential therapeutic targets. These findings underscore the importance of personalized treatment strategies in improving outcomes for osteosarcoma patients.

## 1 Introduction

Osteosarcoma is the most common primary malignant bone tumour, characterized by tissue heterogeneity, local invasion, rapid infiltration and metastasis. It predominantly occurs in the metaphyseal region of long bones, which are areas of rapid bone growth, such as the femur, tibia and humerus [1, 2]. The global incidence of osteosarcoma is 3.4 per million people annually [3], with a higher incidence during adolescence, particularly among those aged 15–19 years [4, 5]. Osteosarcoma has a high mortality and disability rate, making it one of the leading causes of cancer‐related deaths among adolescents [6]. The treatment regimen for osteosarcoma has evolved into a comprehensive approach that primarily includes multi‐agent chemotherapy and limb‐sparing surgery, significantly improving the disease‐free survival rate to 65%–70% [7]. Currently, the combination of high‐dose methotrexate (HD‐MTX), doxorubicin (Adriamycin) and cisplatin is the most effective chemotherapy regimen and is widely used in osteosarcoma treatment. However, chemoresistance and toxic reactions remain major challenges in its application. Future research needs to focus on deeply exploring the mechanisms of chemotherapy, developing new chemotherapy regimens and targeting therapies to overcome these challenges and further improve the prognosis for osteosarcoma patients [8].

To maintain homeostasis, the human body undergoes the death of approximately 50–100 billion cells daily [9]. Based on the triggering mechanisms, cell death is primarily classified into accidental cell death (ACD) and programmed cell death (PCD) [10]. ACD is an uncontrolled biological process, whereas PCD is a finely regulated process that plays a critical role in maintaining tissue homeostasis and eliminating damaged or unnecessary cells. Depending on whether it initiates an adaptive immune response, PCD can be further divided into immunogenic and non‐immunogenic PCD [11]. PCD can be executed through various mechanisms, including apoptosis, autophagy, ferroptosis, pyroptosis, alkaliptosis, cuproptosis, entosis, anoikis, immunogenic cell death, lysosome‐dependent cell death, methuosis, necroptosis, netotic cell death, NETosis, oxeiptosis, parthanatos, and paraptosis [12]. PCD plays a crucial role in the onset, progression and metastasis of malignant tumours. PCD can effectively eliminate damaged or mutated cells, preventing their transformation into cancer cells and inhibiting the proliferation and growth of tumour cells during tumour formation. Additionally, PCD is involved in the regulation and activation of immune cells. By clearing cancer cell debris and releasing cytokines, PCD attracts and activates immune cells, such as T cells and natural killer (NK) cells, thereby enhancing the body's anti‐tumour immune response [13, 14, 15]. Evidence indicates that chemotherapy resistance is mediated by impairing or bypassing PCD mechanisms [16]. An in‐depth understanding and investigation of PCD in tumorigenesis can aid in overcoming chemotherapy resistance and improving the efficacy of cancer treatments.

However, comprehensive analysis of the function of PCD in osteosarcoma is currently limited, and the relationship between PCD and osteosarcoma remains unclear. Therefore, this study employs single‐cell transcriptomics to deeply investigate the effects of chemotherapy on PCD in osteosarcoma cancer cells and the tumour immune microenvironment at the cellular level. Furthermore, we developed a prognostic model named Pyroscore based on pyroptosis‐related genes, offering potential insights for the clinical treatment and management of osteosarcoma.

## 2 Materials and Methods

### 2.1 Data Collection

Bulk RNA sequencing data and clinical information from 88 independent osteosarcoma samples were collected from the TARGET database (https://ocg.cancer.gov/programs/target). Additionally, two osteosarcoma bulk RNA data sets, GSE16091 [17] and GSE39055 [18], along with two single‐cell RNA datasets, GSE152048 [19] and GSE162454 [20], were obtained from the GEO database (https://www.ncbi.nlm.nih.gov/geo/). After screening for typical osteosarcoma samples, 71 bulk RNA sequencing samples and 10 single‐cell transcriptome sequencing samples were selected.

Through literature review, we collected 18 key regulatory genes associated with PCD [21]. These include 580 genes related to apoptosis, 52 genes related to pyroptosis, 88 genes related to ferroptosis, 367 genes related to autophagy, 101 genes related to necroptosis, 19 genes related to cuproptosis, 9 genes related to Parthanatos, 15 genes related to entotic cell death, 8 genes related to netotic cell death, 220 genes related to lysosome‐dependent cell death, 7 genes related to alkaliptosis, 5 genes related to oxeiptosis, 24 genes related to NETosis, 34 genes related to immunogenic cell death, 338 genes related to anoikis, 66 genes related to paraptosis, 8 genes related to methuosis and 23 genes related to entosis. In total, we identified 1078 genes associated with PCD.

### 2.2 Single‐Cell Transcriptome Data Analysis

This study integrated two osteosarcoma single‐cell RNA sequencing data sets from the GEO database (GSE152048 and GSE162454). Raw expression matrices for each sample were batch‐loaded using Read10X() (Seurat version4.2.2), and Seurat objects were constructed via CreateSeuratObject, with filenames assigned as sample identifiers (patient or sample) in the metadata [22]. Corresponding clinical annotation files were then loaded separately and merged with the Seurat object metadata based on sample IDs, with intermediate results saved using qsave. The two data sets were subsequently combined into a unified Seurat object (scRNA) and subjected to comprehensive quality control: percentages of mitochondrial genes (^MT‐), ribosomal genes (^RP[SL]), heat shock proteins (^HSP) and rRNA (^RNA\\d8S5) were calculated, alongside metrics such as log10GenesPerUMI and cell cycle scores. Based on distribution visualizations and statistical thresholds—including nFeature_RNA between 500 and 6000, nCount_RNA > 500, percent.mt < 10% and log10GenesPerUMI > 0.75—cells were rigorously filtered to retain a high‐quality population of 66,486 cells.

The filtered data underwent standard preprocessing: normalization (NormalizeData), identification of highly variable features (FindVariableFeatures), scaling (ScaleData) and principal component analysis (RunPCA). Batch effects were corrected using Harmony (version 1.2.3) integration. The optimal number of principal components was determined by cumulative variance contribution and elbow‐point criteria. UMAP dimensionality reduction and multi‐resolution clustering (resolution = 0.1–0.8) were then performed, with resolution = 0.5 selected for initial clustering. Cell type annotation was conducted using a curated marker gene panel (e.g., epithelial cells, fibroblasts, T and NK cells, macrophages, osteoblastics) sourced from literature and databases; DotPlot visualizations guided manual assignment of the 21 preliminary clusters to 17 biologically meaningful cell types (e.g., cluster “0” → macrophages, “1” → osteoblastics), resulting in a new metadata label first_celltype.

Further analyses explored variations in cell type proportions across samples and in relation to neoadjuvant chemotherapy status, visualized through stacked bar plots, grouped proportional bar charts, and significance‐tested boxplots/violin plots (using *t*‐tests or Wilcoxon tests). Finally, key lineages—myeloid cells (including DCs, macrophages and osteoclasts), T/NK cells, and osteoblastic cells—were isolated for subclustering, with each subset saved as an independent Seurat object to facilitate downstream functional and interaction analyses. The ‘sscVis’ function [23] was employed to analyse the cell type composition preferences before and after chemotherapy, Pathway analysis was performed using the irGSEA (version 3.2.3) package [24], and single‐cell metabolism analysis was conducted with the ‘scMetabolism’ package (version 0.2.1) [25].

### 2.3 Copykat Analysis

To distinguish malignant cells from non‐malignant stromal or immune cells in single‐cell transcriptomic data of osteosarcoma, this study employed the CopyKAT (Copy Number Karyotyping of Tumours) algorithm to infer copy number variations (CNVs) from single‐cell RNA sequencing (scRNA‐seq) data. CopyKAT (version 1.1.0) is a computational tool that identifies tumour cells directly from scRNA‐seq data without requiring matched normal controls [26]. It operates by detecting systematic shifts in gene expression levels across chromosomal regions using a sliding‐window approach, thereby inferring whether cells are diploid (normal) or aneuploid (malignant).

The specific analytical workflow was as follows: First, the raw UMI count matrix was extracted from the Seurat object and used as input for CopyKAT. Key parameter settings included: id.type = “S” (indicating single‐cell sample type), ngene.chr = 5 (requiring at least five genes per chromosome for analysis), win.size = 25 (sliding window size of 25 genes), KS.cut = 0.1 (Kolmogorov–Smirnov test cutoff for determining significant CNVs), Euclidean distance as the distance metric (distance = “euclidean”), and the number of parallel cores set to 1 (n.cores = 1).

The CopyKAT output classified each cell as either “diploid” (presumed non‐tumour/normal cell) or “aneuploid” (presumed malignant tumour cell).

### 2.4 Construction of the Prognostic Model

First, univariate Cox regression was used to identify genes that influence the survival status of osteosarcoma patients (Table S1). Subsequently, a model was developed using an integrated machine learning framework that combines 10 algorithms. This framework includes support vector machine (SVM), least absolute shrinkage and selection operator (Lasso), gradient boosting machine (GBM), random forest, elastic net, stepwise Cox, Ridge, CoxBoost, SuperPC and partial least squares regression with Cox (plsRcox), resulting in a total of 101 algorithm combinations. We set the TARGET‐OS data set as the training set and used two external independent data sets, GSE16091 and GSE39055, as validation sets. The Harrell's concordance index (C‐index) was calculated for all three cohorts. The model combination with the highest C‐index was considered the optimal model. We used the survminer package (version 0.4.9) surv_categorize function to divide the cohorts into high and low Pyroscore groups and evaluated the prognostic significance of Pyroscore using Kaplan–Meier curves. Additionally, calibration curves and receiver operating characteristic (ROC) curves were generated to assess the prognostic performance of Pyroscore.

### 2.5 Differential Gene and Biological Function Enrichment Analysis

Differentially expressed genes between high and low score groups were calculated using the “DESeq2” package (version 1.38.3), with *p*‐values adjusted for multiple testing using the Benjamini–Hochberg (BH) procedure and the selection criteria set at *p*‐value < 0.05 and |log2FoldChange| > 0.5. To further identify functional differences between the high and low score groups, we performed gene ontology (GO), Kyoto Encyclopedia of Genes and Genomes (KEGG), Reactome, and GSEA pathway enrichment analyses using the ‘clusterProfiler’ package (version 4.6.2) [27], ‘ReactomePA’ (version 1.42.0) [28] and ‘GSEABase’ (version 1.60.0) [29].

### 2.6 Immune Microenvironment Analysis

To comprehensively evaluate the tumour immune microenvironment in patients with two different statuses, we employed a variety of algorithms from the IBOR package (version 0.99.9) [30] including single‐sample GSEA (ssGSEA), cell type identification by estimating RNA transcript relative subpopulations (CIBERSORT), Tumour Immune Estimation Resource (TIMER), Immunophenoscore (IPS), Microenvironment Cell Populations‐counter (MCP‐counter), EPIC, xCELL and quanTIseq. These methods allowed us to accurately assess differences in immune cell composition between different patients. To further evaluate which cells are correlated with Pyroscore, we used the cor.test () function to analyse immune cell subpopulations associated with the score. Finally, we assessed tumour purity, immune score and stromal score in different patients using the ESTIMATE package (version 1.0.13) [31].

### 2.7 Chemotherapy Drug Sensitivity Analysis

We utilized the ‘pRRophetic’ R package (version0.5) [32] to calculate the half‐maximal inhibitory concentration (IC_50_) of 82 chemotherapy drugs in high and low score groups. The Wilcoxon method was used to evaluate differences in drug sensitivity between the two groups.

### 2.8 Statistical Analysis

All statistical analyses were performed using R software. To evaluate the impact of risk factors on survival outcomes, Cox regression models and Kaplan–Meier (KM) survival analyses were used. Differences in continuous variables were compared using the Wilcoxon rank‐sum test (for non‐normally distributed and heteroscedastic samples) or the *t*‐test (for normally distributed and homoscedastic samples). Pearson rank correlation analysis was employed to assess the correlation between two continuous variables. A *p*‐value less than 0.05 was considered statistically significant.

## 3 Results

### 3.1 Identification of Key Osteosarcoma Cell Subpopulations During Chemotherapy Using Single‐Cell Analysis

**FIGURE 1:**
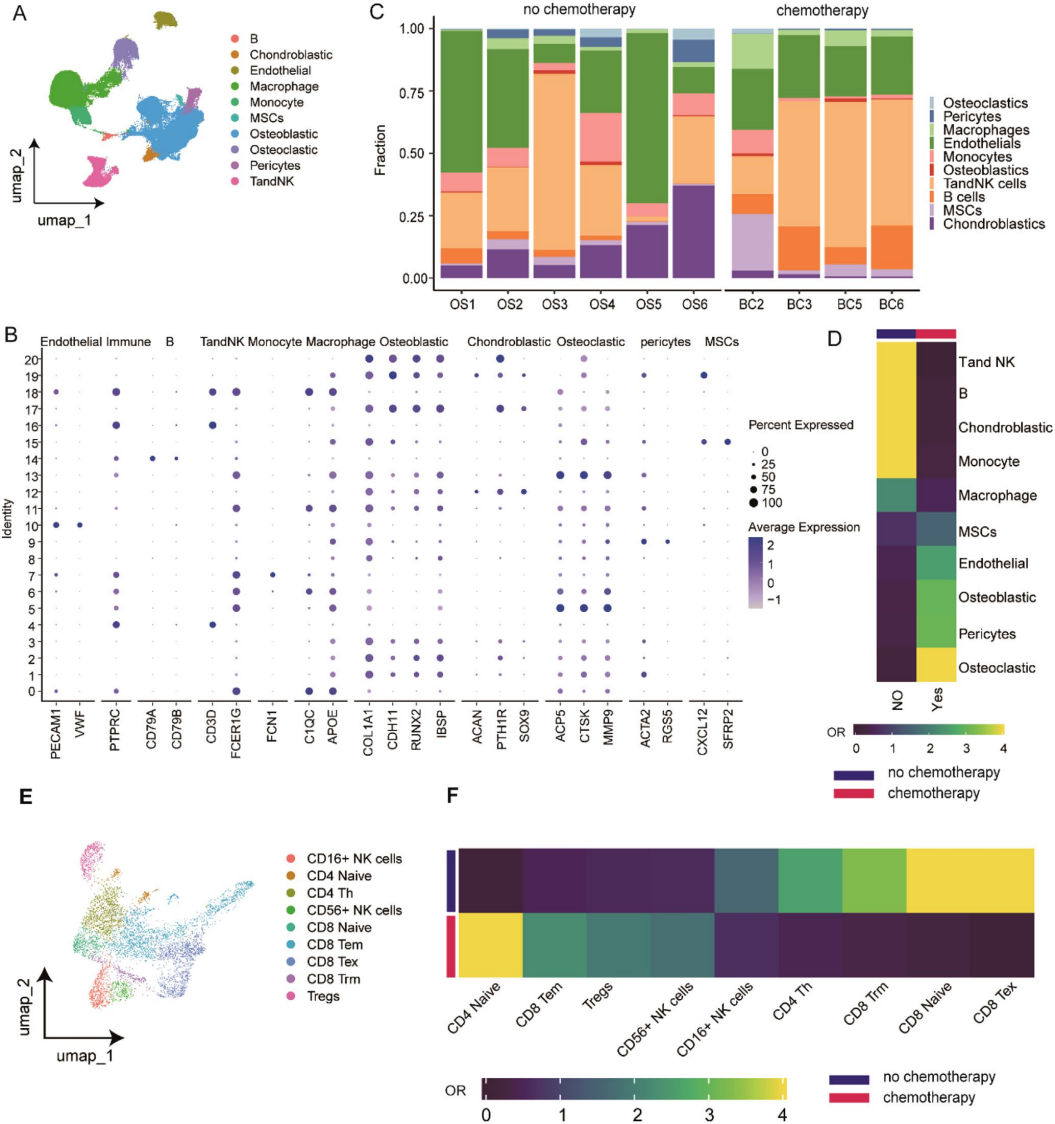
Single‐cell sequencing reveals chemo‐interactive cell subpopulations in osteosarcoma. (A) Cell types identified by osteosarcoma single‐cell data. (B) Marker gene dot plots used to annotate single‐cell data (endothelial cells: PECAM1, VWF; immune cells: PTPRC; B cells: CD79A, CD79B; T and NK cells: CD3D, FCER1G; monocytes: FCN1; macrophages: C1QC, APOE; Osteoblasts: COL1A1, CDH11, RUNX2, IBSP; Osteoclasts: ACAN, PTH1R, SOX9; Osteoblasts: ACP5, CTSK, MMP9; Pericytes: ACTA2, RGS5; MSCs: CXCL12, SFRP2). (C) Histogram of cell ratios between chemo and non‐chemo patients. (D) Demonstration of cellular OR heatmap between chemo‐ and non‐chemo‐treated patients, OR > 1.5 represents cellular subtypes are more inclined to be distributed in the corresponding patients. (E) T‐cell subpopulation re‐annotated UMAP downscaled heatmap. (F) T‐cell subpopulation OR heatmap between chemo and non‐chemo‐treated patients.

We conducted stringent quality control on the single‐cell data, removing low‐quality cells that did not meet our criteria, resulting in a dataset of 27,406 cells from 10 samples (Table S2). Notably, we integrated two independent datasets that exhibited batch effects; hence, we employed the Harmony algorithm to significantly reduce non‐biological batch effects. Subsequently, through normalization and dimensionality reduction clustering, we categorized the cells into 21 subpopulations. Based on marker genes for each cell subtype, these subpopulations were further annotated into 10 cell clusters (Figure 1A,B). The cell proportion bar plots and OR index indicated that under chemotherapy, the proportions of osteoclasts, endothelial cells, mesenchymal stem cells and pericytes increased, while the proportions of T and NK cells, B cells, chondroblasts, monocytes and macrophages decreased (Figure 1C,D). We then performed further sub‐clustering of T cells (Figure 1E). In T cells, chemotherapy led to an upregulation in the proportions of CD4^+^ naive T cells, CD8 effector memory T cells, regulatory T cells and CD56^+^ NK cells, while the proportions of CD16^+^ NK cells, CD4 helper T cells, CD8 tissue‐resident memory T cells, CD8 naive T cells and CD8 exhausted T cells decreased (Figure 1F).

### 3.2 Identification of Chemotherapy‐Related Pathways in Key Cell Populations via Single‐Cell Analysis

**FIGURE 2:**
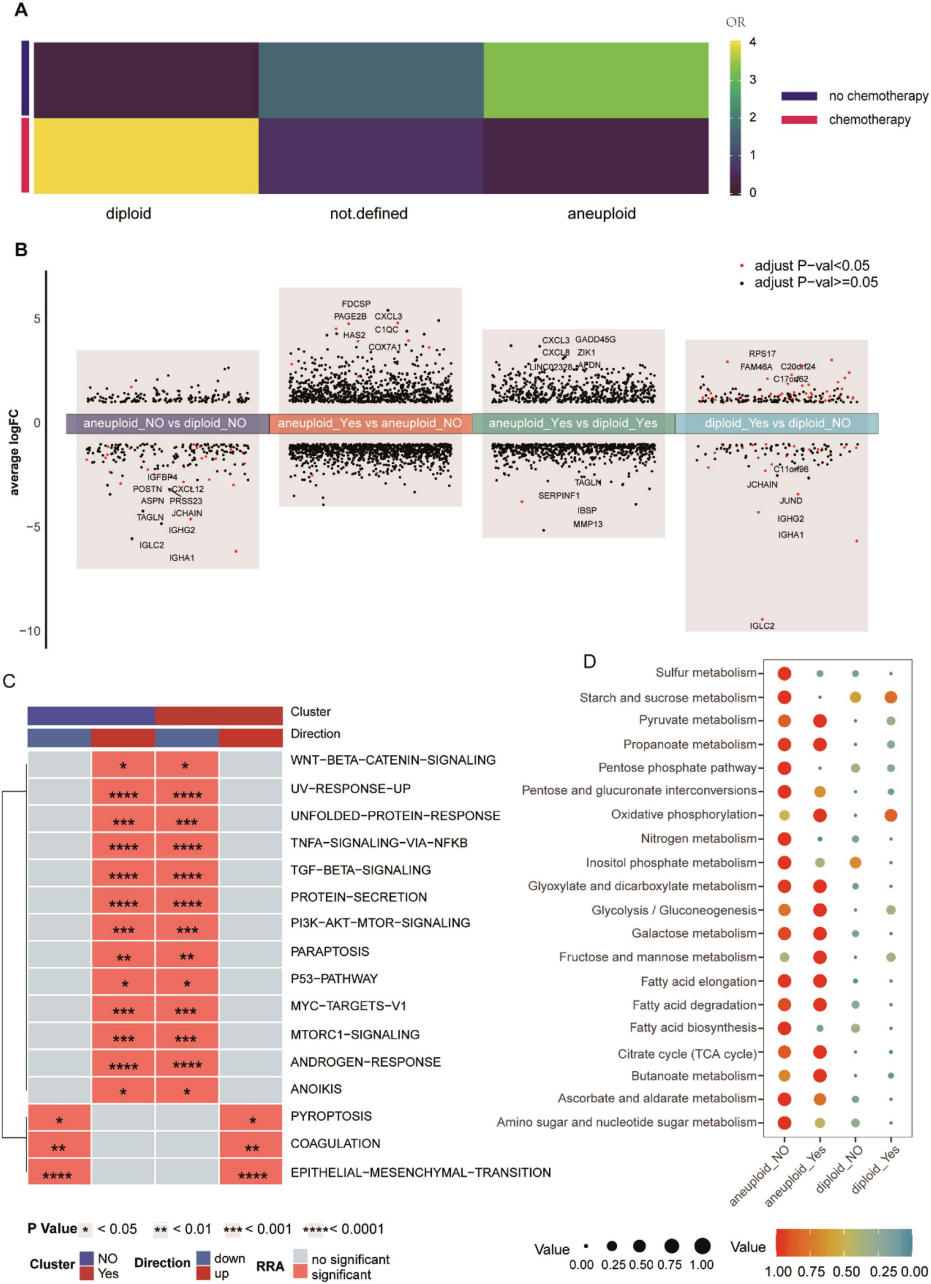
Single cell analysis to identify functional differences as well as metabolic differences due to chemotherapy. (A) Heatmap of OR between aneuploidy and aneuploidy between chemotherapy and non‐chemotherapy patients. (B) Volcano heatmap for demonstrating differential genes between aneuploidy and aneuploidy and between chemotherapy and non‐chemotherapy patients. (C) IrGSEA heatmap for demonstrating functional differences between chemotherapy and non‐chemotherapy patients. (D) Dot plot for demonstrating metabolic differences between aneuploidy and aneuploidy and between chemotherapy and non‐chemotherapy patients.

First, using the copykat package, we inferred normal cells (diploid) and tumour cells (aneuploid) within osteoblasts. As expected, the proportion of tumour cells in chemotherapy patients significantly decreased compared to non‐chemotherapy patients (Figure 2A). We then performed differential gene expression analysis across various groups, including normal cells (diploid), malignant cells (aneuploid) and chemotherapy status. The analysis revealed significant downregulation of immunoglobulin, periostin, insulin‐like growth factor binding protein 4 and TALGN expression, while CXCL3 and CXCL8 were significantly upregulated (Figure 2B). To further explore the impact of chemotherapy on tumour pathways and PCD, we utilized irGSEA for sample evaluation. irGSEA integrates multiple enrichment scoring methods, including AUCell, UCell, singscore, ssGSEA, JASMINE, and viper and employs the robust rank aggregation (RRA) algorithm for comprehensive assessment. The results indicated a significant upregulation of the pyroptosis pathway in tumour cells of chemotherapy patients (Figure 2C), suggesting that chemotherapy may induce PCD in tumour cells by activating pyroptosis. Single‐cell metabolic analysis showed a significant increase in the metabolic levels of malignant cells compared to normal cells; however, chemotherapy significantly inhibited sulphur metabolism, starch and sucrose metabolism, pentose phosphate pathway, inositol phosphate metabolism, nitrogen metabolism and fatty acid metabolism (Figure 2D). These findings suggest that chemotherapy may inhibit tumour cell proliferation by suppressing certain carbohydrate and sulphur metabolic pathways [11, 12].

### 3.3 Construction of an Osteosarcoma Prognostic Model Based on Pyroptosis‐Related Genes

**FIGURE 3:**
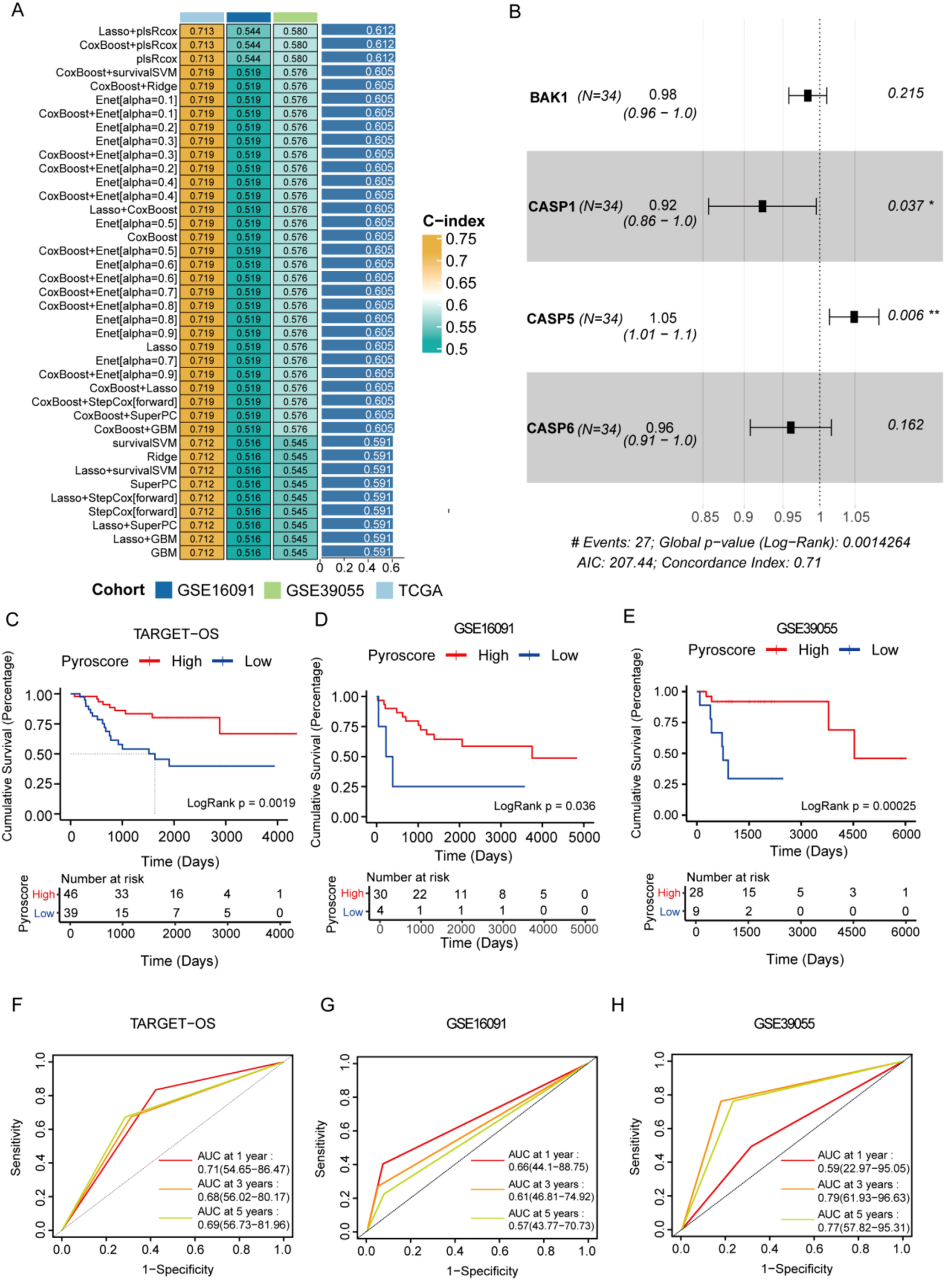
Prognostic significance of Pyroscore. (A) C‐index values of 101 combinations in three data sets of TARGET‐OS, GSE16091, and GSE39055 using 10 machine‐learning algorithms. (B) Multifactorial COX regression analyses to assess the association of the four genes with prognosis. (C–E) Plotting of TARGET‐OS, GSE16091, and GSE39055 by Pyroscore, GSE39055 survival curves for both high and low groups. (F–H) ROC curves for 1, 3, and 5 years in each data set.

Based on single‐cell transcriptome analysis, we found that chemotherapy can activate pyroptosis in tumour cells. Consequently, we constructed an osteosarcoma prognostic model based on pyroptosis‐related genes. Univariate Cox regression analysis identified 11 pyroptosis‐related genes, which were used to establish a model within a machine‐learning framework. Interestingly, the algorithms Lasso+plsRcox, CoxBoost+plsRcox and plsRcox identified the same optimal model (Figure 3A). This model comprises four genes: BCL2 Antagonist/Killer 1 (BAK1), caspase‐1 (CASP1), caspase‐5 (CASP5), and caspase‐6 (CASP6) (Figure 3B). Patients were divided into high and low score groups based on the Pyroscore. Kaplan–Meier analysis showed that patients in the high score group had significantly better survival than those in the low score group (Figure 3C–E). TimeROC analysis results showed that in the TARGET‐OS data set, the 3‐year AUC values were 0.71, 0.68 and 0.69; in the GSE16091 data set, the 3‐year AUC values were 0.66, 0.61 and 0.57; and in the GSE39055 data set, the 3‐year AUC values were 0.59, 0.79 and 0.77 (Figure 3F–H). These results highlight the excellent predictive performance and clinical value of the Pyroscore.

### 3.4 Functional Differences Between High and Low Pyroscore Groups

**FIGURE 4:**
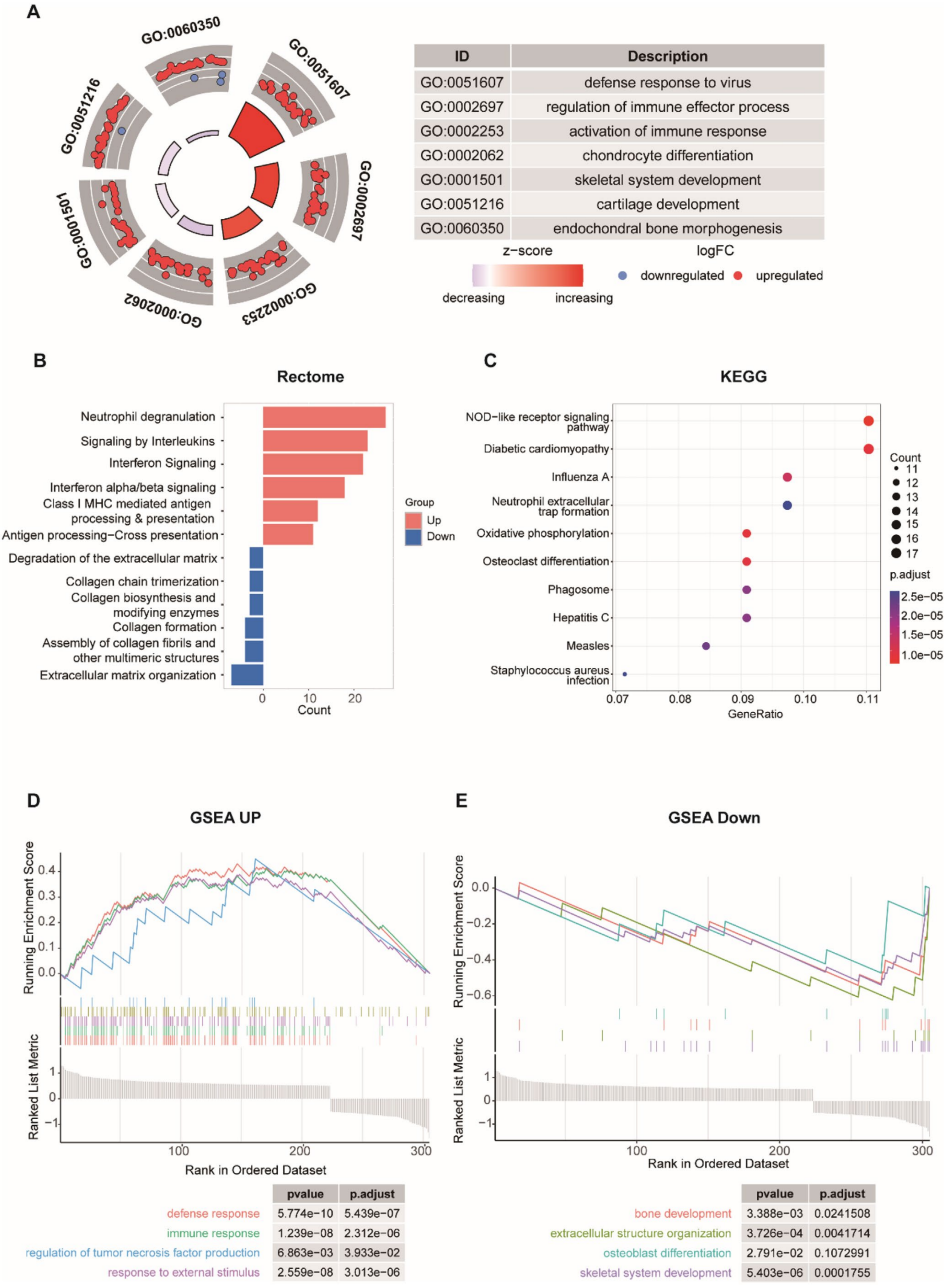
Functional differences between Pyroscore groups. (A) Circle plot of GO enrichment analysis of differential genes in Pyroscore high and low groups. (B) Histogram of rectome enrichment analysis of differential genes in Pyroscore high and low groups. (C) Dot plot of KEGG enrichment analysis of differential genes in Pyroscore high and low groups. (D, E) Results of GSEA enrichment analysis of differential genes in Pyroscore high and low groups.

To gain deeper insights into the functional differences between the high and low Pyroscore groups, we first performed differential gene expression analysis. Using |logFoldChange| > 0.5 and *p*‐value < 0.05 as criteria, we identified 223 upregulated genes and 82 downregulated genes. Gene ontology enrichment analysis indicated that immune system and defence‐related pathways were activated in the high Pyroscore group, while pathways related to cartilage and skeletal system development and differentiation were potentially inhibited (Figure 4A). Reactome enrichment results showed that upregulated genes were enriched in immune‐related signalling pathways such as neutrophil degranulation, interleukin signalling, antigen presentation and interferon signalling. Downregulated genes were mainly enriched in pathways related to extracellular matrix organization, collagen formation, collagen fibril organization and collagen biosynthesis (Figure 4B). KEGG enrichment results showed that the relevant genes were enriched in the pathways of osteoclast differentiation, neutrophil extracellular trap formation, oxidative phosphorylation, NOD‐like receptor signalling pathway, diabetic cardiomyopathy, influenza A, phagolysosomes, hepatitis C, measles and *Staphylococcus aureus* infection (Figure 4C). GSEA enrichment analysis of differentially expressed genes further revealed that upregulated pathways were primarily involved in responses to external stimuli, regulation of tumour necrosis factor production, immune responses and defence responses (Figure 4D). Downregulated pathways were mainly associated with skeletal system development, osteoblast differentiation, extracellular structure organization and skeletal development. In summary (Figure 4E). we believe that the better prognosis of the high Pyroscore group may be attributed to the activation of the immune system and the inhibition of pathways related to the skeletal system, cartilage tissue and extracellular matrix.

### 3.5 Differences in Immune Infiltration Between High and Low Pyroscore Groups

**FIGURE 5:**
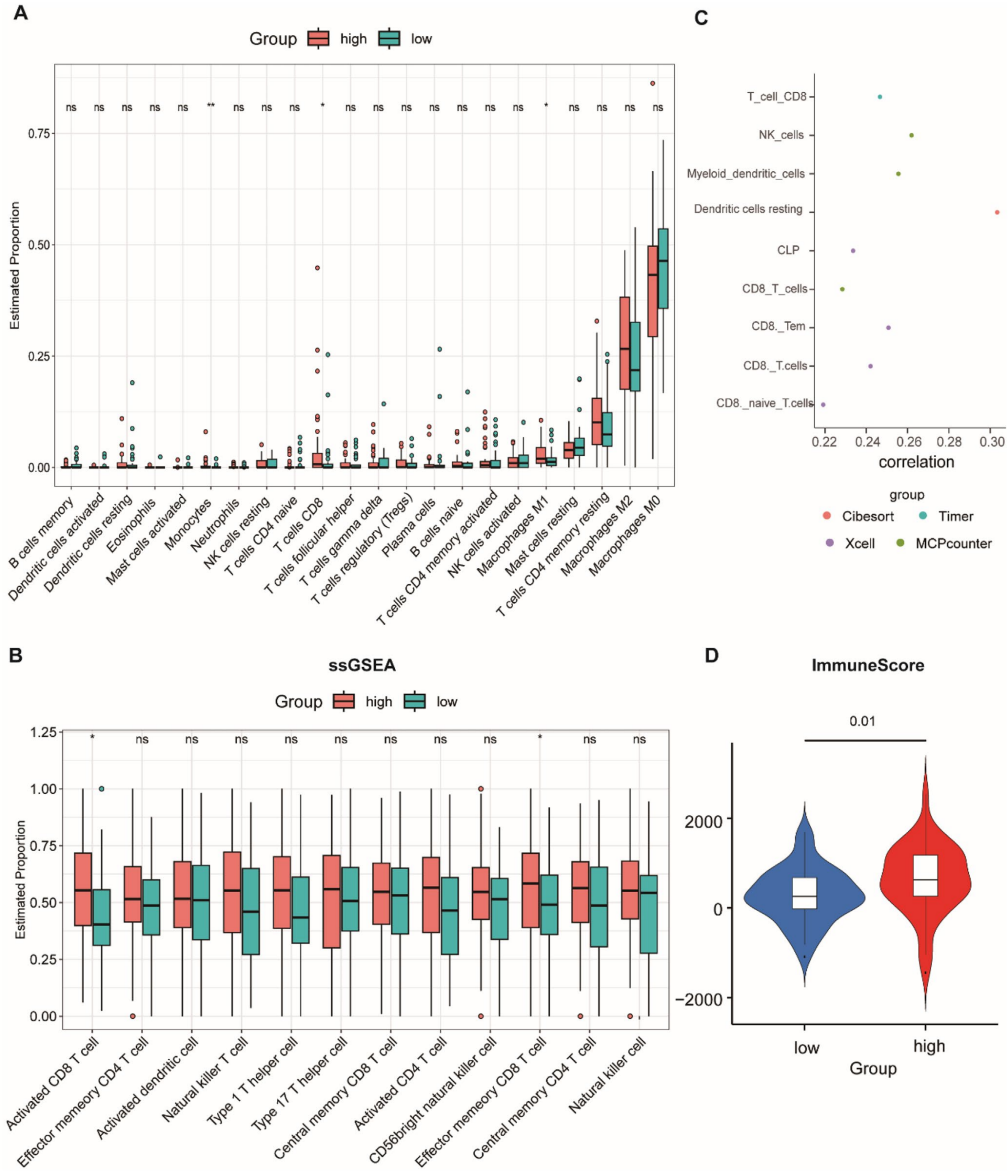
Tumour immune microenvironment analysis between patients with high and low Pyroscore. (A) Histogram of the results of the cibesort immune infiltration analysis. (B) Ssgsea analysis of the infiltration of antitumour‐associated immune cells between the two clans. (C) Correlation of immune cells with Pyroscore. (D) ESTIMATE assessment of immune scores of the two groups with high and low Pyroscore.

We evaluated the differences in the tumour immune microenvironment between the high and low Pyroscore groups. First, we analysed immune infiltration between the two groups using seven immune algorithms. CIBERSORT analysis revealed that the proportions of M1 macrophages and CD8 T cells were significantly higher in the high Pyroscore group compared to the low Pyroscore group (Figure 5A). To further identify which immune cell subpopulations were significantly enriched in the high Pyroscore group, we used the ssGSEA algorithm to evaluate 28 immune cell types. We found that cancer‐related immune cells, such as activated CD8^+^ T cells and effector memory CD8 T cells, were significantly more abundant in the high score group than in the low score group (Figure 5B). Subsequent correlation analysis across seven immune algorithms showed that CD8 T cells were significantly positively correlated with Pyroscore (Figure 5C), and the immune scores were significantly higher in the high Pyroscore group than in the low Pyroscore group (Figure 5D). These findings suggest that the high Pyroscore group exhibits greater anti‐tumour immune cell infiltration, with CD8 T cells likely playing a crucial role in the better prognosis observed in this group.

### 3.6 Prediction of Chemotherapy Drug Sensitivity

**FIGURE 6:**
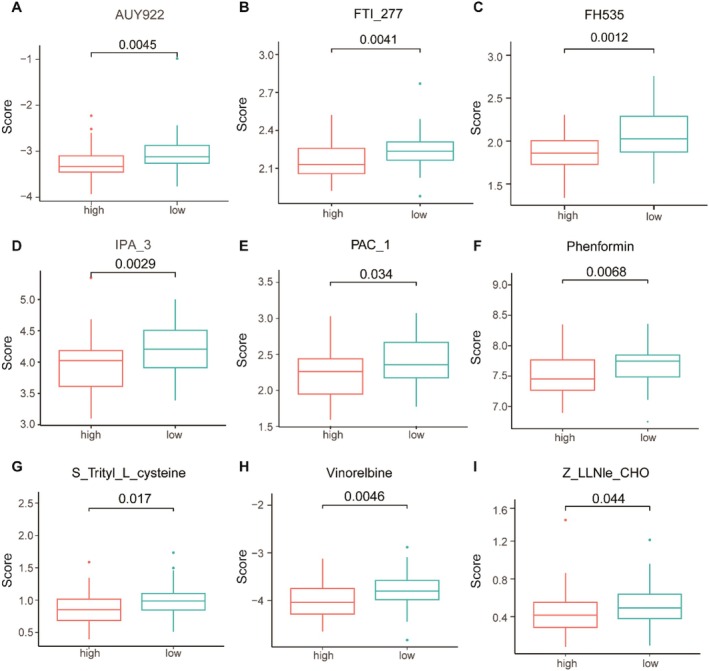
Screening of chemotherapeutic agents for the treatment of osteosarcoma. (A–I) IC_50_ of AUY922 (A), FTI‐277 (B), FH535 (C), IPA‐3 (D), PAC‐1 (E), Phenformin (F), S‐Trityl‐L‐cysteine (G), Vinorelbine (H), and Z‐LLNle‐CHO (I) drugs were assessed to determine the drug sensitivity between the high and low Pyroscore groups.

To predict chemotherapy drug sensitivity, we used the pRRophetic algorithm to estimate the chemotherapy response of osteosarcoma patients based on the half‐maximal inhibitory concentration (IC_50_) available in the Cancer Genome Project (CGP) database. In our study, nine small molecule compounds showed significantly different responses between the high and low Pyroscore groups, including AUY922 (*p* = 0.0045), FTI‐277 (*p* = 0.0041), FH535 (*p* = 0.0015), IPA‐3 (*p* = 0.0029), PAC‐1 (*p* = 0.035), Phenformin (*p* = 0.0068), S‐Trityl‐L‐cysteine (*p* = 0.017), Vinorelbine (*p* = 0.0045) and Z‐LLNle‐CHO (*p* = 0.044). The low Pyroscore group was more sensitive to these chemotherapy drugs (Figure 6A–I). These small molecule drugs may be potential therapeutic agents for osteosarcoma patients with poor prognosis (Table S3).

## 4 Discussion

Osteosarcoma is a highly malignant bone tumour with limited efficacy in traditional chemotherapy. Neoadjuvant chemotherapy is currently the best treatment option. However, over the past 30 years, there has been little progress in developing new effective treatment regimens [33, 34, 35]. Therefore, an in‐depth study of the mechanisms of osteosarcoma cell response to chemotherapeutic drugs could lead to the development of more effective treatment strategies and improve patient survival rates. Single‐cell transcriptome technology provides gene expression information at the cellular level, which helps to reveal tumour heterogeneity, analyse the tumour microenvironment, elucidate gene expression patterns of cancer cells, and discover new targets and biomarkers. Single‐cell transcriptome data provide a powerful tool for osteosarcoma research, offering deep and broad insights at the molecular and cellular levels [36, 37, 38, 39].

This study utilized single‐cell analysis to identify key osteosarcoma cell subpopulations and their responses to chemotherapy, revealing critical insights into cellular and molecular dynamics during treatment. By applying rigorous quality control and batch effect removal algorithms, we integrated chemotherapy‐treated and untreated osteosarcoma samples, providing a robust data set with a detailed landscape of the osteosarcoma microenvironment. Chemotherapy can induce dynamic changes in the tumour microenvironment. The proportion of aneuploid tumour cells significantly decreased in chemotherapy‐treated patients. Our findings provide new evidence at the single‐cell level for the efficacy of chemotherapy. However, since chemotherapeutic drugs are not selective, immune cells also decrease post‐chemotherapy, necessitating close monitoring and management to maintain or restore immune function, reduce complications and improve patient quality of life. Further subpopulation classification revealed an increase in CD8 effector memory T cells and a decrease in CD8 exhausted T cells post‐chemotherapy. T cell exhaustion leads to reduced effector functions, preventing cytotoxic CD8 T cells from controlling tumour progression at advanced stages [40]. These findings suggest that chemotherapy can induce T cell reprogramming, reactivating their tumour‐killing effects.

Pyroptosis is a newly discovered form of PCD characterized by the activation of inflammasomes and subsequent cleavage of gasdermin, leading to cell membrane rupture and the release of cellular contents [41]. Substantial evidence indicates that pyroptosis affects tumour development [42, 43, 44]. Our study found that the pyroptosis pathway was significantly upregulated in the tumour cells of chemotherapy patients, indicating that chemotherapy induces PCD through pyroptosis. Pyroptosis also alters the tumour immune microenvironment, enhancing immune responses and inhibiting tumour progression [45]. Furthermore, single‐cell metabolic analysis showed that chemotherapy significantly inhibited key metabolic pathways, including sulphur metabolism, starch and sucrose metabolism, the pentose phosphate pathway, inositol phosphate metabolism, nitrogen metabolism and fatty acid metabolism. The inhibition of these pathways also suggests that chemotherapy prevents tumour cell proliferation [46].

Our study leveraged single‐cell transcriptome analysis to reveal that chemotherapy activates pyroptosis in tumour cells, elucidating the role of pyroptosis in osteosarcoma. To explore the clinical significance of pyroptosis‐related genes, we constructed a prognostic model based on these genes. Notably, the Lasso+plsRcox, CoxBoost+plsRcox and plsRcox algorithms all yielded the same optimal model, emphasizing its robustness and reliability. The model includes four key genes: BCL2 Antagonist/Killer 1 (BAK1), Caspase 1 (CASP1), Caspase 5 (CASP5) and Caspase 6 (CASP6), which play crucial roles in the pyroptosis pathway. BAK1, a pro‐apoptotic member of the BCL‐2 protein family, promotes apoptosis in response to cellular stress and damage, playing an important role in tumour suppression [47, 48]. However, BAK1 has been considered a poor prognostic factor in some studies of hepatocellular carcinoma and head and neck squamous cell carcinoma [49, 50]. Our results suggest that BAK1 also appears to be a poor prognostic factor for osteosarcoma. CASP1, CASP5 and CASP6 belong to the caspase family, which has dual roles in tumours: inhibiting tumour growth by inducing apoptosis of tumour cells and promoting tumour growth by inducing inflammatory responses [51, 52, 53, 54].

This study has several limitations that warrant acknowledgment. First, the inference of key biological processes—such as pyroptosis—is primarily based on indirect evidence derived from transcriptomic data and has not been directly validated by functional experiments (e.g., protein‐level detection of pyroptosis markers or morphological assessment). Consequently, these conclusions remain hypothetical and require further experimental confirmation. Second, drug sensitivity predictions were generated using the pRRophetic algorithm, which relies on pharmacological response data from in vitro cancer cell lines. However, substantial differences exist between cell lines and actual tumour tissues in terms of genetic background, heterogeneity and drug metabolism, limiting the clinical generalizability of these predictions; thus, they should not be equated directly with real‐world patient treatment responses. Additionally, although an external validation cohort was included, its sample size was relatively small and derived from a single centre, potentially compromising the generalizability of our findings. Prospective, multicentre studies with larger cohorts are therefore urgently needed to further validate the robustness and clinical applicability of our results.

In summary, our study, through single‐cell transcriptomic analyses, elucidated the impacts of chemotherapy on the osteosarcoma microenvironment and hindered osteosarcoma progression by activating pyroptosis pathways. Our prognostic model highlights the significance of pyroptosis‐related genes in osteosarcoma outcomes. Furthermore, by applying Pyroscore to classify osteosarcoma into molecular subtypes, we disclosed stark immune characteristic disparities and functional state diversities among these subtypes. Our findings underscore the critical importance of immune system activation and optimization for improving patient outcomes in osteosarcoma. By expanding the range of chemotherapeutic options for osteosarcoma and underpinning precision medicine, our research paves the way for tailored chemotherapy in clinical practice, with the potential to elevate treatment efficacy and reduce patient burdens. These discoveries offer fresh perspectives and directions for future osteosarcoma treatment strategies.

## Author Contributions

**Ziyang Yu:** methodology. **Guoyong Yu:** writing – review and editing, data curation, supervision. **Tao Jin:** writing – original draft, methodology. **Lei Dong:** writing – original draft, data curation, visualization. **Wang Kai:** writing – original draft. **Weifeng Liu:** writing – review and editing, data curation, supervision.

## Funding

This work was supported by the National Natural Science Foundation of China (52473310), the Natural Science Foundation of Beijing (L252065), the Beijing Municipal Health Commission (BJRITO‐RDP‐2025) and the Beijing Xisike Clinical Oncology Research Foundation (Y‐HR2020QN‐0413).

## Conflicts of Interest

The authors declare no conflicts of interest.

## Supporting information


**Table S1:** Clinical metadata for transcriptomics. Clinical and survival information of patients from three osteosarcoma transcriptomic data sets (GSE16091, GSE39055 and GSE152048).
**Table S2:** Clinical metadata for single‐cell. Pathological and clinical characteristics of patients in single‐cell osteosarcoma cohorts.
**Table S3:** Summary of small‐molecule compounds. List of small‐molecule compounds used in functional screening, including drug names, targets and mechanisms of action.

## Data Availability

All the data used in this project can be obtained from the TARGET‐OS and GEO databases.
